# Sero-prevalence of specific *Leptospira* serovars in fattening pigs from 5 provinces in Vietnam

**DOI:** 10.1186/s12917-017-1044-1

**Published:** 2017-05-08

**Authors:** Hu Suk Lee, Nguyen Viet Khong, Huyen Nguyen Xuan, Vuong Bui Nghia, Hung Nguyen-Viet, Delia Grace

**Affiliations:** 1 International Livestock Research Institute, Regional Office for East and Southeast Asia, Room 301-302, B1 Building, Van Phuc Diplomatic Compound, 298 Kim Ma Street, Ba Dinh District, Hanoi Vietnam; 2 National Institute of Veterinary Research, 86 Truong Chinh, Phuong Mai, Dong Da, Hanoi Vietnam; 3 International Livestock Research Institute, Nairobi, Kenya

**Keywords:** Leptospirosis, Vietnam, Pig, Serovar, Microscopic agglutination test (MAT)

## Abstract

**Background:**

Leptospirosis is a zoonotic bacterial disease with a worldwide distribution. In Vietnam, leptospirosis is considered endemic. In pigs, leptospirosis can result in reproductive problems (such as abortion and infertility) which lead to economic loss. In addition, transmission to people presents a public health risk. In Vietnam, few national studies have been conducted on sero-prevalence of leptospirosis in pigs. The main objective of this study was to evaluate the sero-prevalence and incidence of presumptive infective *leptospira* serovars in fattening pigs from 5 provinces in Vietnam.

**Results:**

Blood samples from fattening pigs were randomly collected at slaughterhouses. We collected 1959 sera samples from 5 provinces (Son La, Hanoi, Nghe An, Dak Lak and An Giang) between January and early June 2016. The microscopic agglutination test (MAT) was used to identify the serogroups/serovars. Overall, the sero-prevalence was 8.17% (95% CI: 6.99–9.47) and serovar Tarassovi Mitis (2.19%) had the highest prevalence followed by Australis (1.94%), Javanica (1.68%) and Autumnalis (1.17%) using a cutoff (≥ 1:100). The sero-prevalence among female pigs (5.28%, 95% CI: 3.94–6.93) was slightly higher than among male pigs (4.88%, 95% CI: 3.51–6.58), but this difference was not statistically significant.

**Conclusions:**

Leptospirosis in pigs may be a useful indicator of the human/animal burden in Vietnam and a risk assessment tool. The presence of some of the identified serovars suggests that wildlife may play an important role in the transmission of leptospirosis to domesticated pigs in Vietnam. Therefore, strengthened monitoring and surveillance systems are needed to better understand the epidemiology of the disease and prevent or reduce infection in humans and animals.

## Background

Leptospirosis is a zoonotic bacterial disease with a worldwide distribution [1–4]. It is caused by more than 250 pathogenic serovars of the genus *Leptospira* [5]. More than 150 mammals can be infected with *Leptospira* spp. Reservoir hosts include rodents, wild animals and domestic animals (such as cattle, pigs and dogs) [6–10]. *Leptospira interrogans* serovar Bratislava has been associated with reproductive problems in pigs (such as abortion, infertility and birth of weak piglets), which lead to economic loss [11, 12]. In addition, pigs are considered to be reservoir hosts for serovars Muenchen, Pomona and Tarassovi Mitis and can also be infected with Icterohaemorrhagiae from rats, Canicola from dogs and Hardjo from cattle [13–19].

In Vietnam, leptospirosis was first identified in 1930, and the country is considered endemic with a peak during the rainy season [5, 20–22]. In 2012, a total of 241 rats were collected in the Mekong delta in Vietnam and of those, 18.3% tested positive for *Leptospira* serovars [19]. The identified serovars with the highest prevalence were *Rattus norvegicus* (33.0%), *Bandicota indica* (26.5%) and *Rattus tanezumi* (24.6%). It is assumed that rats are important reservoirs of the disease. In 2002, a study conducted in the Mekong Delta found that the seroprevalence in sows was 73% using a cut-off of 1:100. The serovar with the highest prevalence was Braitsalva (52%). Although leptospirosis is a notifiable disease in humans in Vietnam, only a few cases have been reported to the centralized system. However, previous studies show that leptospirosis is a public health concern and is often responsible for 20% of acute fevers of unknown origin [22–25]. A study conducted among healthy people in the Mekong Delta identified the following positive serovars: Bataviae (21.7%), Panama (15.2%), Icterohaemorrhagiae (13.7%) and Australis (8.7%) [24].

To our knowledge, few studies have been conducted to evaluate the sero-prevalence of leptospirosis in pigs in Vietnam. Therefore, the main objective of this study was to evaluate the sero-prevalence and incidence of presumptive infective *Leptospira* serovars in fattening pigs from 5 provinces in Vietnam.

## Methods

Blood samples from fattening pigs (6–9 months old and weighing 60–120 kg) were randomly collected from the jugular vein at slaughterhouses from 5 provinces (Son La, Hanoi, Nghe An, Dak Lak and An Giang) between January and early June 2016. The selected provinces were representative of the different ecological and climatic zones in Vietnam. To evaluate the sero-prevalence of serovars in each province, the research team confirmed that all pigs sampled were raised in the respective province and not imported from other provinces.

All blood samples were immediately placed in cool boxes at slaughterhouses. Sera were extracted after centrifugation and stored at −20 °C at local laboratories until they were transported to the National Institute of Veterinary Research (NIVR) in Hanoi for analysis. The sample size was calculated based on 50% prevalence, a precision level of 5 and 95% confidence interval using STATA. This indicated at least 385 samples per province were to be collected using multi-stage sampling [province (5), district (25), commune (125)]. For each province, a total of 25 communes (5 communes per district) were selected from 5 districts, based on the availability of pig slaughterhouses. Within each commune, 15–16 samples were randomly collected from more than one slaughterhouse. However, we were not able to achieve the sample size in some communes due to lack of time and resources. Additional information (date of collection, sampling area and sex) was collected using a check-list.

**Fig. 1:**
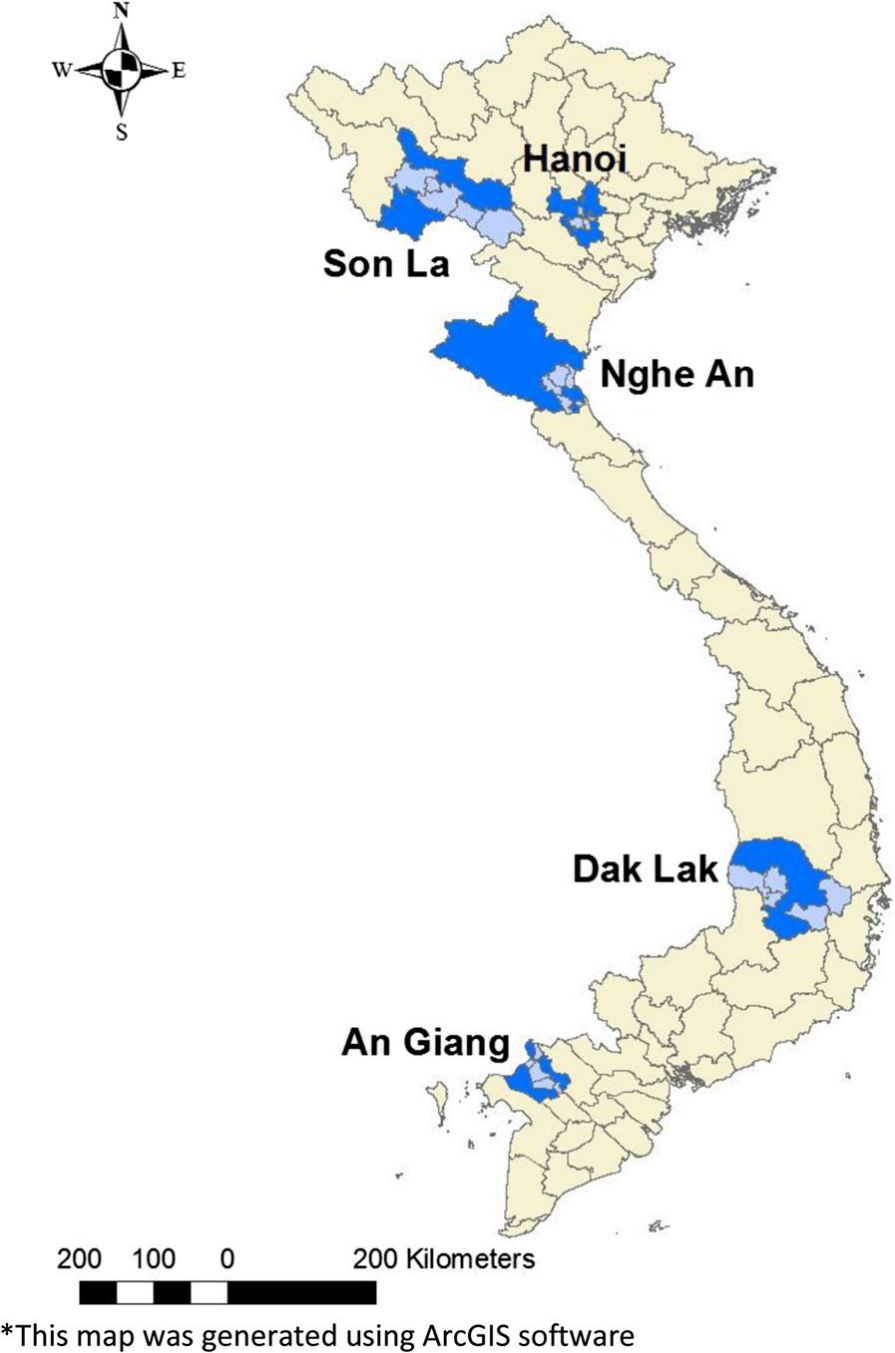
Selected sampling districts (*light blue*) within each province (*dark blue*). *This map was generated using ArcGIS software

The microscopic agglutination test (MAT) was used to identify the serogroups/serovars using two-fold serial dilutions of serum in testing, starting with 1:100 up to 1:800. Each serovar result was recorded as the highest dilution of the serum which showed at least 50% agglutination of live leptospires compared to the control sample. Positive tests were defined as MAT results ≥1:100 for at least one of the 15 serovars (Table 1). A logistic regression model was constructed with the cluster effect (by having commune as a random effect) to evaluate the association between the sex and positivity when controlling for age (6–9 months old). The logistic regression model was assessed for goodness-of-fit using the Hosmer-Lemeshow test [26]. The results were expressed as Odds ratio (OR) and 95% confidence interval (CI). An alpha level of <0.05 was set for statistical significance. All data were recorded in Microsoft Excel 2010 and analyzed using STATA (version 14.0, StataCorp, College Station, TX, USA). ArcGIS version 10.3.1 (ESRI, Redlands, CA, USA) was used to generate the map (Fig. 1).

**Table 1:** List of *Leptospira* antigens used in the MAT

No.	Genomospecies	Serogroup	Serovar	Strain
1	*L. interrogans*	Australis	Australis	Ballico
2	L. interrogans	Autumnalis	Autumnaliss	Akiyami A
3	L. interrogans	Bataviae	Bataviae	Van Tienen
4	L. interrogans	Canicola	Canicola	Hond Utrech IV
5	L. kirschneri	Grippotyphosa	Grippotyphosa	Moskva V
6	L. interrogans	Hebdomadis	Hebdomadis	Hebdomadis
7	L. interrogans	Icterohaemorrhagiae	Icterohaemorrhagiae	Verdun
8	L. borgpetersenii	Javanica	Javanica	Veldrat Batavia 46
9	L. noguchii	Panama	Panama	CZ214K
10	L. interrogans	Pomona	Pomona	Pomona
11	L. interrogans	Pyrogenes	Pyrogenes	Salinem
12	L. borgpetersenii	Sejroe	Hardjo	Hardjo Bovis
13	L. borgpetersenii	Sejroe	Saxkoebing	Mus 24
14	*L. biflexa*	Semaranga	Patoc	Patoc I
15	L. borgpetersenii	Tarassovi	Tarassovi	Mitis Johnson

Reference: http://www.kit.nl/biomedical-research/product-category/leptospira-strains/

## Results

**Fig. 2:**
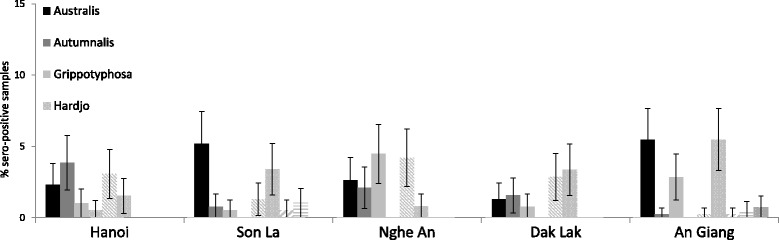
Percentage with 95% confidence interval of sero-positive samples by serovar in each province using cutoff titer ≥1:100

We collected 1959 sera samples from 5 provinces. However, seven samples from Nghe An and three samples from Son La showed hemolysis and, were discarded from the study. We detected reactions against nine different serovars. Overall, the sero-prevalence was 8.17% (95% CI: 6.99–9.47). Yen Chau district in Son La province had the highest sero-prevalence. Hanoi province (9.49%) had the highest sero-prevalence whereas Dak Lak province (7.01%) had the lowest sero-prevalence, but the differences were not statistically significant (Table 2). The most frequently detected presumptive infective serogroup was Tarassovi Mitis (2.19%), followed by Australis (1.94%), Javanica (1.68%), Autumnalis (1.17%) and Grippotyphosa (1.07%) using a cutoff titer of ≥1:100 (Table 3). Considering province and serovars Autumnalis, Australis, Grippotyphosa, Javanica and Australis had the highest sero-prevalences in Hanoi, Son La, Nghe An, Dak Lak and An Giang, respectively (Fig. 2). By using a low cutoff titer (≥ 1:100), the percentage of positive MAT results for Leptospira serovars was highest for Tarassovi Mitis (2.20%) and Australis (1.94%) (Table 3). A total of 6 samples were positive for two or more serovars: one sample with three serovars (Australis, Grippotyphosa and Javanica) and five samples with two serovars.

**Table 2:** Sero-prevalence (%) with 95% CI for *Leptospira* serovars in fattening pigs in Vietnam using MAT

Province (no.)	District (no.)	Sero-positive samples (a titer ≥ 1:100 for any serovars)	Sero-positive (%) with 95% CI (each district)	Sero-positive (%) with 95% CI (each province)
Hanoi (390)	Chuong My (57)	4	7.01 (1.94–17.00)	9.49 (6.77–12.84)
	Hoai Duc (56)	9	16.07 (7.62–28.33)	
	Thanh Oai (106)	7	6.60 (2.70–13.13)	
	Thanh Tri (90)	6	6.67 (2.49–13.95)	
	Dan Phuong (81)	11	13.58 (6.98–23.00)	
Son La (384)	Mai Son (77)	4	5.19 (1.43–12.77)	7.03 (4.68–10.07)
	Moc Chau (77)	1	1.30 (0.03–7.02)	
	Son La (76)	2	2.63 (0.332–9.18)	
	Thuan Chau (76)	2	2.63 (0.332–9.18)	
	Yen Chau (77)	18	23.38 (14.48–34.41)	
Nghe An (380)	Dien Chau (73)	7	9.59 (3.94–18.76)	8.68 (6.05–11.98)
	Nam Dan (68)	8	11.76 (5.22–21.87)	
	TP. Vinh (95)	8	8.42 (3.71–15.92)	
	Yen Thanh (67)	3	4.48 (0.93–12.53)	
	Do Luong (77)	7	9.09 (3.73–17.84)	
Dak Lak (385)	Buon Don (81)	5	6.17 (2.03–13.82)	7.01 (4.67–10.04)
	Cu Mgar (77)	3	3.90 (0.81–10.97)	
	Krong Bong (67)	4	5.97 (1.65–14.59)	
	M’Drak (79)	11	13.92 (7.16–23.55)	
	Buon Me Thuot (81)	4	4.94 (1.36–12.16)	
An Giang (384)	Chau Phu (80)	9	11.25 (5.28–20.28)	9.38 (6.65–12.74)
	Chau Thanh (85)	8	9.41 (4.15–17.71)	
	Chau Doc (84)	3	3.57 (0.74–10.08)	
	Long Xuyen (83)	8	9.64 (4.25–18.11)	
	Tan Chau (88)	8	9.09 (4.01–17.13)	
Total	1959	160	8.17 (6.99–9.47)	8.17 (6.99–9.47)

**Table 3:** MAT results for *Leptospira* serovars in pigs by using 4 cutoff titers during the study period

Sero-positive results									
Serovar	Total tested samples (n)	≥ 1:100		≥ 1:200		≥ 1:400		≥ 1:800	
		*n*	% (95% CI)	*n*	% (95% CI)	*n*	% (95% CI)	*n*	% (95% CI)
Tarassovi Mitis	1959	43	2.20 (1.55–2.84)	11	0.56 (0.23–0.89)	3	0.15 (0–0.33)	1	0.05 (0–0.15)
Australis	1959	38	1.94 (1.37–2.65)	16	0.82 (0.42–1.22)	7	0.36 (0.09–0.62)	4	0.20 (0.001–0.40)
Javanica	1959	33	1.68 (1.11–2.25)	8	0.41 (0.13–0.69)	3	0.15 (0–0.33)	1	0.05 (0–0.15)
Autumnalis	1959	23	1.17 (0.75–1.76)	7	0.36 (0.09–0.62)	2	0.10 (0–0.24)	1	0.05 (0–0.15)
Grippotyphosa	1959	21	1.07 (0.62–1.53)	9	0.46 (0.16–0.76)	4	0.20 (0.001–0.40)	4	0.20 (0.001–0.40)
Canicola	1959	2	0.10 (0–0.24)	1	0.05 (0–0.15)	0	Null	0	Null
Hebdomadis	1959	2	0.10 (0–0.24)	1	0.05 (0–0.15)	0	Null	0	Null
Icterohaemorrhagiae	1959	2	0.10 (0–0.24)	2	0.10 (0–0.24)	2	0.10 (0–0.24)	1	0.05 (0–0.15)
Hardjo	1959	1	0.05 (0–0.15)	1	0.05 (0–0.15)	0	Null	0	Null

The sero-prevalence among female pigs (5.28%, 95% CI: 3.94–6.93) was slightly higher than among male pigs (4.88%, 95% CI: 3.51–6.58). The logistic regression model showed that there was no statistically significant difference between male and female (Table 4). The Hosmer-Lemeshow goodness of fit test showed that there was evidence of poor fit.

**Table 4:** Logistic regression model of leptospirosis with random effect (positive was considered if cutoff titer ≥1:100)

Variable	Category	Adjusted Odds ratio	95% CI	*P*-value
Sex	Female	Reference	N/A	N/A
	Male	1.09	0.77–1.55	0.624

*CI* confidence interval, *NA* not applicable as reference group

**Fig. 3:**
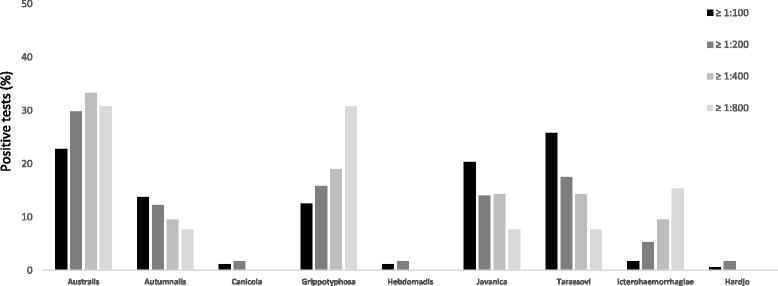
Percentage of positive MATs by Leptospira serovar using 4 different cutoff titers during the study period

Among positive serovars using a different cutoff [1:100 (no. 167), 1:200 (no. 57), 1:400 (no. 21) and 1:800 (no. 13)], the proportion of serovars Australis (except for 1:800 titer), Grippotyphosa and Icterohaemorrhagiae increased as the cutoff titer increased, whereas it decreased for Autumnalis, Javanica and Tarassovi (Fig. 3).

## Discussion

This large, national study was conducted to assess the sero-prevalence of specific *Leptospira* serovars in pigs across the country. Overall, the sero-prevalence was lower than reported in previous studies (ranging from 13 to 79%) [27–30]. This may be because our samples were collected during the dry and early wet seasons when sero-prevalence is likely to be lower than in the rainy season [31]. Also, most of the previous studies were conducted in the Mekong delta in southern Vietnam that is relatively close to river areas, which might provide more opportunities to come into contact with contaminated water. In addition, samples were collected from slaughterhouses and so represent relatively young animals (pigs age: 6–9 months). One study suggested that older pigs were more likely to be exposed to *Leptospira* [28]. Moreover, animals sent for slaughter may be less likely to be visibly ill. We found that Tarassovi Mitis, Australis, Javanica, Autumnalis and Grippotyphosa had relatively high positive rates which were similar to those reported by the previous studies [27, 28]. Pigs are considered a reservoir for serovar Tarassovi Mitis which had the highest positive rate in our study, but one study showed that Pomona had the highest rate [32]. Another study found that Bratislava had the highest sero-prevalence among sows in the Mekong Delta of Vietnam, but it was not included in our analysis [28]. Serovars Australis, Grippotyphosa, Tarassovi Mitis were detected in our study, and other studies also found these serovars in wild boars [33–35]. In France, a re-emergence of brucellosis in outdoor pigs was linked to contact with wild boars [36]. It is possible that wild boars may play an important role in the transmission of leptospirosis to domesticated pigs in Vietnam. In addition, we detected serovars Javanica and Icterohaemorrhagiae, implying that rats may have infected pigs [19]. Other studies found that increased movement of wild animals during fall and early winter, associated with seeking shelter and food for the winter season, increases contact with domestic animals or organisms shed by wild animals [35, 37]. Further investigations are required to better understand the role of wildlife in transmission of leptospirosis in Vietnam.

Leptospirosis in humans is a notifiable disease in Vietnam (one of 28 notifiable diseases) that should be reported to the preventive medicine networks. However, due to lack of medical facilities and public awareness, few human cases have been reported in Vietnam. Only 48 cases were officially reported between 2008 and 2013 (annual incidence rate: 0.011 per 100,000). Surveys typically find higher levels; one study conducted in Tien Giang (Mekong Delta region) found a sero-prevalence of 18.8% among 1400 and the most prevalent serovars were Bataviae, Panama, Icterohaemorrhagiae and Australis [24]. A serological study conducted among children in southern Vietnam in 2003 reported a sero-prevalence of 12.8% [25].

Leptospirosis is considered an occupational risk for agriculture workers, mining workers, sewer maintenance workers, veterinarians and other individuals who are likely to come into contact with contaminated water or soil and infected animals [4].

Serovars Autumnalis, Australis, Grippotyphosa and Javanica have been recognized as major causes of human leptospirosis in Asian countries, and were detected in our study. It is possible that the disease could be transmitted from pigs to humans [20, 38, 39]. In Vietnam, small-scale pig production accounts for 80% of the total production, providing opportunities for transmission from pigs to farmers and their families [40]. In the United States of America, an epidemiological study showed that swine producers and slaughterhouse workers had relatively higher sero-prevalence of leptospirosis in comparison with other occupational groups [41]. In Mexico, keeping domestic livestock (cattle and pigs) significantly increased the odds of disease in a rural community [42]. Therefore, swine sero-prevalence may be useful as a surrogate marker of the human/animal leptospirosis incidence in Vietnam. Additional studies could evaluate the sero-prevalence in agricultural farmers and butchers as well as identifying the modes of transmission and potential risk factors through surveys of farm workers.

In particular, potential environmental risk factors (such as climate factors, rural or urban status and proximity to lakes and rivers) should be evaluated. Previous studies showed a seasonal pattern and a positive association with the amount of rainfall [31, 36, 43]. In Vietnam, there are two distinct seasons: dry (from January to May) and wet (from June to December). It could be possible that outbreaks of leptospirosis are more likely to occur during the wet season in Vietnam. Therefore, strengthened monitoring and surveillance systems are needed to better understand the epidemiology of leptospirosis and prevent or reduce infection in humans and animals.

## Conclusion

Leptospirosis in pigs may be a useful indicator of the human/animal burden in Vietnam and a risk assessment tool. These studies are a way of providing evidence to veterinarians on diagnosis, vaccination and control of zoonotic pathogens. In addition, the presence of some of the identified serovars suggests that wildlife may play an important role in the transmission of leptospirosis to domesticated pigs in Vietnam. Further investigation is needed in each region into the possible role of different wildlife species or environmental conditions in contributing to infection.

## Abbreviations

CI: Confidence interval
MAT: Microscopic agglutination test
NIVR: National institute of veterinary research
